# Focus on Mesenchymal Stem Cell-Derived Exosomes: Opportunities and Challenges in Cell-Free Therapy

**DOI:** 10.1155/2017/6305295

**Published:** 2017-12-19

**Authors:** Lin Cheng, Kun Zhang, Shuying Wu, Manhua Cui, Tianmin Xu

**Affiliations:** ^1^Gynecology and Oncology Department of the Second Hospital of Jilin University, Ziqiang Street 218, Changchun 130000, China; ^2^Medical Research Center, Second Clinical College, Jilin University, Ziqiang Street 218, Changchun 130000, China

## Abstract

Mesenchymal stem cells have been at the forefront of regenerative medicine for many years. Exosomes, which are nanovesicles involved in intercellular communication and the transportation of genetic material transportation that can be released by mesenchymal stem cells, have been recently reported to play a role in cell-free therapy of many diseases, including myocardial infarction, drug addiction, and status epilepticus. They are also thought to help ameliorate inflammation-induced preterm brain injury, liver injury, and various types of cancer. This review highlights recent advances in the exploration of mesenchymal stem cell-derived exosomes in therapeutic applications. The natural contents, drug delivery potency, modification methods, and drug loading methods of exosomes are also discussed.

## 1. Introduction

Mesenchymal stem cells (MSCs) originate from the mesoderm of many tissues, including bone marrow, liver, spleen, peripheral blood, adipose, placenta, and umbilical cord blood, and have the capacity to self-renew and the ability to generate differentiated cells. Over the last decade, MSCs have emerged as a popular research topic because of their potential role in regenerative medicine, immunoregulation, neuroprotection, and antitumor effects originally attributed to direct cell replacement. However, experimental data indicates that most MSCs are largely cleared, while a small proportion will integrate into injured tissue after intravenous injection [1]. Furthermore, the “cell replacement theory” does not account for the sufficient durations in a variety of disease models [2, 3]. Recently, several mechanisms have been put forward regarding the therapeutic potential of MSCs, including (1) paracrine factors involving proteins/peptides and hormones and (2) the transfer of mitochondria or exosomes/microvesicles packaging multitudinous molecules [4].

Exosomes are a family of nanoparticles with a diameter in the range of 40–100 nm that are generated inside multivesicular endosomes or multivesicular bodies (MVBs) and are secreted when these compartments fuse with plasma membrane [5]. Exosomes are enriched in endosome-derived components as well as many bioactive molecules such as proteins, lipids, mRNAs, microRNAs (miRNAs), long noncoding RNAs (lncRNAs), transfer RNA (tRNA), genomic DNA, cDNA, and mitochondrial DNA (mtDNA) [6–12]. It has also been reported that exosomes may be released from multiple cell types, including reticulocytes [13], immunocytes, tumor cells, and MSCs [14]. This suggests that the secretion of exosomes is a general cellular function that plays an important role in the intercellular transfer of information.

In this review, we focus on the mechanisms of exosomes/microvesicles, covering the current knowledge on biological characteristics and their potential cell-free therapeutic applications for MSC-derived exosomes.

## 2. Characterization and Isolation of Exosomes

Exosomes were first discovered by Harding's group as “a garbage can” in maturing sheep reticulocytes [13]. Originally, they were thought to have a typical “cup-shaped” or “saucer-like” morphology when analyzed by electron morphology [15, 16]. Zabeo's group revealed a wide diversity in exosome morphology when purified from homogeneous cell types (the human mast cell line HMC-1). They classified exosome morphology into nine categories: (1) single vesicle; (2) double vesicle; (3) triple vesicle or more; (4) small double vesicle; (5) oval vesicle; (6) small tubule; (7) large tubule; (8) incomplete vesicle; and (9) pleomorphic vesicle [17]. This categorization suggested that different morphologies of exosomes may be accompanied by various and specific functions. Exosomes also contain surface proteins unique to the endosomal pathway, which are generally used to characterize exosomes and distinguish them from microvesicles (MVs), apoptotic bodies, and other vesicles (Table 1), such as tetraspanins (CD63, CD81, and CD9), heat shock proteins (Hsc70), lysosomal proteins (Lamp2b), the tumor-sensitive gene 101 (Tsg101), and fusion proteins (CD9, flotillin, and annexin) [12, 18]. Exosomes are released in almost all types of extracellular fluids, including blood, urine, amniotic fluid, ascites, hydrothorax, saliva, breast milk, seminal fluid, and cerebrospinal fluid. Exosomal content greatly depends on cellular origin. For example, exosomes derived from B lymphocytes that bring functional MHCI, MHCII, and T cell costimulatory molecules can stimulate T cell proliferation [19]. Furthermore, cancer cell-derived exosomes contain gelatinolytic enzymes and other cell adhesion-related molecules to help tumor progression and metastasis [20]. Importantly, these cancer cell-derived exosomes are actively incorporated by MSCs in vitro and in vivo, in that the transfer of exosomal proteins and miRNAs acquire the physical and functional characteristics of tumor-supporting fibroblasts [21, 22]. For more details on the molecular cargos and extracellular signal transmission pathway of exosomes, the reader may refer to ExoCarta (http://www.exocarta.org) or EVpedia (http://evpedia.info), as well as the American Society for Exosomes and Microvesicles (http://www.asemv.org), for an in-depth exploration.

Ultracentrifugation and a commercial kit rooted in polymer-based precipitation are the most well-established purification protocols [16]. Other conventional validated isolation methods described in the literature include ultrafiltration, chromatography, and affinity capture [23]. New protocols have been established in order to facilitate the large-scale and high-purity manufacture of exosomes. Microfluidic techniques [24] are based on electrochemical, electromechanical, viscoelastic [25], optical, nonoptical, and other principles, yet the isolation is a mixed population of small nanoparticles without further demonstration of their intracellular origin. Thus, we use the term exosomes in this review to refer to extracellular vesicles characterized by exosome-specific surface markers, regardless of the primitive appellations in the published data.

## 3. Cargos and Functions of MSC-Derived Exosomes

**Figure 1:**
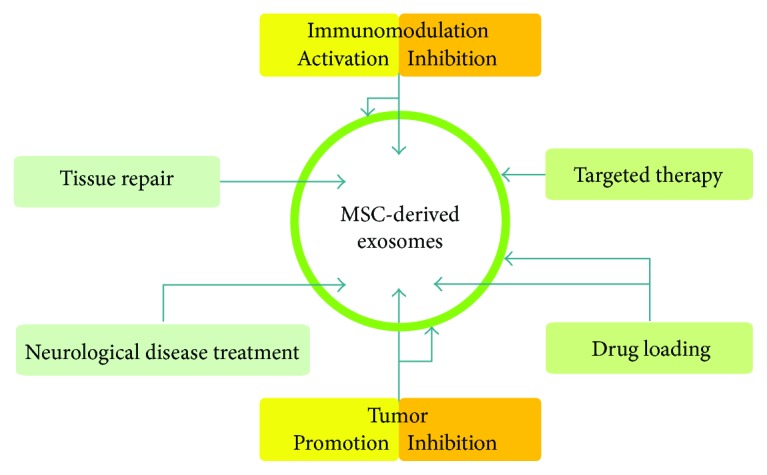
The main functions of MSC-derived exosomes. The external bilayer (green circle) is the membrane and the internal bilayer (white circle) is packed with various bioactive compounds.

The abundance of cargos identified from MSC-derived exosomes attracts broad attention because of their therapeutic potential in cardiovascular disease, tissue (kidney, liver, skin, and cornea) repair, immune disease, tumor inhibition, and neurological disease (Figure 1). They function largely via the constant transfer of miRNAs and proteins, resulting in the alteration of a variety of activities in target cells via different pathways.

### 3.1. Protein

Over 900 species of proteins have been collected from MSC-derived exosomes according to ExoCarta. With the exception of some common proteins involved in cell metabolism and the cytoskeleton, many proteins have been found in different tissue sources of MSC-derived exosomes. Proteomic studies by Kang's group identified 103 proteins from neural stem cell-derived exosomes. For example, the presence of polymyositis/scleroderma autoantigen 2 (PM/Scl2), a highly specific nuclear autoantigen, indicates that exosomes may be involved in triggering autoimmunity. They also found an imparity between exosomes larger than the baseline (50 nm) and those of smaller morphology [26]. These findings may explain the phenomenon recently observed by Caponnetto et al. regarding size-dependent cellular uptake of exosomes by target cells [27]. Intriguingly, all enzymes involved in the ATP synthesis of glycolysis (glyceraldehyde 3-phosphate dehydrogenase (GAPDH), phosphoglycerate kinase (PGK), phosphoglucomutase (PGM), enolase (ENO), and pyruvate kinase m2 isoform (PKm2)), as well as the rate-limiting glycolytic enzyme phosphorylated PFKFB3 that upregulates phosphofructose kinase, were identified in MSC-derived exosomes. Furthermore, oxidative stress was reduced via peroxiredoxins and glutathione S-transferases in MSC-derived exosomes [28], which suggests that replenishing glycolytic enzymes to increase ATP production, as well as additional proteins to reduce oxidative stress through exosomal transportation, may help reduce cell death in myocardial ischemia/reperfusion injury. Comparable levels of VEGF, extracellular matrix metalloproteinase inducer (EMMPRIN), and MMP-9 have also been reported in MSC-derived exosomes. These three proteins play a vital role in stimulating angiogenesis [29], which could be fundamental for tissue repair. Recent experimental evidence summarized by Burrello suggests that transcriptional factors, such as Nanog, octamer-binding transcription factor 4 (Oct-4), HoxB4, and Rex-1, play an important role in the immune system [30]. For example, HoxB4 has been shown to affect DC maturation and T-cell proliferation, differentiation, and activation through WNT signaling. Interestingly, membrane proteins and exosome-specific surface markers, such as CD81, CD63, and CD9, may affect the immune response by regulating cell adhesion, motility, activation, and signal transduction [31]. Several studies have also shown that exosomes derived from MSCs harbor cytokines and growth factors, such as TGF*β*1, interleukin-6 (IL-6), IL-10, and hepatocyte growth factor (HGF), which have been proven to contribute to immunoregulation [30].

### 3.2. miRNAs

miRNAs consist of a class of small noncoding RNAs that regulate gene expression posttranscriptionally by targeting mRNAs to induce suppression of protein expression or cleavage [32]. Many miRNAs have been found in MSC-derived exosomes and are reportedly involved in both physiological and pathological processes such as organism development, epigenetic regulation, immunoregulation, tumorigenesis, and tumor progression. Notably, exosomes with membrane structure act as preservers and deliverers of miRNAs, transferring functional miRNAs into recipient cells. It has been reported that exosomal miR-23b, miR-451, miR-223, miR-24, miR-125b, miR-31, miR-214, and miR-122 [33, 34] may inhibit tumor growth and stimulate apoptosis through different pathways. For instance, miR-23b promotes dormancy in metastatic breast cancer cells via the suppression of the target gene MARCKS, which encodes a protein that promotes cell cycling and motility [34]. MiR-16, shuttled by MSC-derived exosomes, has also been found to suppress angiogenesis by downregulating VEGF expression in breast cancer cells [35]. Recently, let-7f, miR-145, miR-199a, and miR-221, which are released from umbilical MSC-derived exosomes, have been found to largely contribute to the suppression of hepatitis C virus (HCV) RNA replication [36]. Di Trapani's group evaluated the immunomodulatory effects exerted by MSC-derived exosomes on unfractionated peripheral blood mononuclear cells and purified T, B, and NK cells. They observed that exosomes had higher levels of miRNAs compared to MSCs and could also induce inflammatory priming via increasing levels of miR-155 and miR-146, which are two miRNAs involved in the activation and inhibition of inflammatory reactions [37]. Similar immunosuppressive functions have also been reported in animal experiments by Cui et al. [38]. Exosomes from MSCs effectively increased the level of miR-21 in the brain of AD mice. Additionally, replenishment of miR-21 restored the cognitive deficits in APP/PS1 mice and prevented pathologic features by regulating inflammatory responses and restoring synaptic dysfunction [38]. Recent studies have also shown that aging is substantially controlled by hypothalamic stem cells, partially through the release of exosomal miRNAs [39]. However, contradictions regarding these outcomes remain. A quantitative analysis of exosomal miRNA abundance and stoichiometry by Chevillet's group quantified both the number of exosomes and the number of miRNA molecules in replicate samples isolated from diverse sources. Regardless of the source, the study indicated that, on average, over 100 exosomes would need to be examined to observe one copy of a given abundant miRNA, suggesting that most individual exosomes do not carry biologically significant numbers of miRNAs and are thus unlikely to be functional individually as vehicles for miRNA-based communication [40].

### 3.3. Others

In 2006, MSC-derived exosomes that could modulate the phenotype of target cells, supporting self-renewal of hematopoietic progenitors and multipotency by transfer of growth factors and mRNA, were first reported. For instance, exosomal SOX2 was found to initiate innate responses against microbial infection through neutrophil activation [41]. Although MSC-derived exosomes have the same morphology as exosomes from other cells and carry typical markers, they are quite different in regard to compartmentalization and protein and RNA composition. For example, studies have indicated that not all MSC-derived exosomes are equivalent [42]. Baglio's group [6] characterized the small RNAome of exosomes released by early passage adipose-MSCs (ASC) and bone marrow-MSCs (BMSCs). They found a large discrepancy in the proportion of miRNAs in total small RNA content between cells (19–49%) and exosomes (2–5%), suggesting that the miRNAs in exosomes do not merely reflect the cellular content. Further studies regarding the overrepresentation of small RNA content-tRNAs revealed a similar outcome. The most abundant tRNA in ASC exosomes, tRNA GCC (Gly), represented only a small fraction (5%) of the total cellular tRNA. Importantly, the authors also determined that the striking differences in tRNA species seemed to be associated with the differentiation status of MSCs. Recent research has shown that the stability of exosome composition is susceptible to localized environmental conditions. For example, hypoxia and inflammatory signals, such as lipopolysaccharides, may be strong interference factors [43].

## 4. Exosomes as Drug Delivery Vehicles

Optimal features of drug delivery vehicles may be applied to improve carrier qualities, including cellular tropism, efficient therapeutic cargos, appropriate physicochemical properties, and sufficient immune tolerance. Among the many drug platforms, liposomes have been the preferred pharmaceutical vehicles for drug delivery. A wide range of liposome products have been approved for the treatment of diseases, including fungal infections, pain management, hepatitis A, influenza, and various types of cancer [44, 45]. In contrast to liposomes, exosomes are optimal for drug delivery because of their natural properties and plasticity with minor modifications. Here, we compare exosomes and liposomes and suggest that exosomes may be a promising star for drug delivery.

### 4.1. Exosomes versus Liposomes

Exosomes and liposomes are both coated with a phospholipid membrane. The membrane structure of exosomes is inlayed with multiple natural biomolecules, such as surface proteins and MHCs, while liposomes may be modified with targeting ligands or inert polymeric molecules such as oligosaccharides, glycoproteins, polysaccharides, and synthetic polymers [45]. The size of liposomes is in the range of 30 nm to several microns [46]. Smaller liposomes (as small as exosomes) display a prolonged circulation time compared to larger ones, but the capacity for optimal drug reservation and release profiles is partly lost. For more details regarding circulation time and biodistribution, readers can refer to other sources [46]. Regarding cellular interactions and uptake, liposomes can be equipped with targeting ligands, which can bind to receptors or other molecules that are specific or overexpressed by target cells for interactions and the intracellular delivery of drugs [46]. However, the drug delivery of liposomes is not efficient, since many modifications have been designed to minimize clearance and poisonousness. In general, liposomes accumulate in the macrophages of the liver and spleen after intravenous injection. Few liposomes are interspersed in other tissues, which may be due to the lack of immunocompatibility. On the other hand, exosomes are born with many features of an ideal drug delivery vehicle. For example, they exhibit lower toxicity compared to liposomes. In addition, they are well tolerated by the immune system, even across the blood-brain barrier, avoiding phagocytosis or degradation by macrophages [47]. Exosomes exhibit an innate targeting tendency. For instance, MSC-derived exosomes home preferentially to inflamed tissues and tumor tissues [48]. Furthermore, abundant bioactive materials within exosomes or on the surface provide primitive treatment potential, and there are abundant modification methods for membrane targeting and drug loading. Alvarez-Erviti et al. engineered dendritic cells to express Lamp2b, an exosomal membrane protein, fused to the neuron-specific RVG peptide and loaded these modified exosomes with siRNAs by electroporation. These intravenously injected exosomes showed a strong knockdown of BACE1 (mRNA (60%) and protein (62%)), a therapeutic target of Alzheimer's disease, in wild-type mice [49].

### 4.2. Exosomal Modification and Cargo Loading

To amplify the therapeutic effects, many studies try to modify and load various treatment factors into exosomes via various methods. To date, these methods can be classified into two categories: (1) loading after isolation and (2) loading exosomes during biogenesis.

The first approach has been applied to load chemotherapeutic agents, siRNAs, and miRNAs. To reduce immunogenicity and toxicity of doxorubicin, Tian's group facilitated exosomal tumor targeting by engineering mouse immature dendritic cells (imDCs) to express a well-characterized exosomal membrane protein (Lamp2b) fused to a breast cancer-specific iRGD peptide (CRGDKGPDC). Chemotherapeutic agents were loaded via electroporation. The results showed an encapsulation efficiency of up to 20% and exosomal-delivered doxorubicin specific to breast cancer cells in vitro, leading to strong antiproliferative activity without overt toxicity after intravenous injection of BALB/c nude mice [50]. For nucleic acid, electroporation method has also been the first-rank used reported in several studies [49, 51]. Although these studies provided positive delivery outcomes, debates remain. Some studies indicate that siRNA encapsulation is an illusion caused by nonspecific aggregate formation, independent of the exosomes. In addition, no significant encapsulation of siRNA could be measured when aggregate formation was blocked [52]. Therefore, it is necessary to establish multiple protocols for loading exosomes with nucleic acid.

The second approach is based on transfection methods to package active proteins, nucleic acid, and other active molecules into exosomes, where cells are transfected with an engineered effector-expressing vector. Liu's group used this method to load cells with opioid receptor Mu (MOR) siRNA in order to treat drug addiction via downregulating the expression of MOR, the primary target for opioid analgesics used clinically, including morphine, fentanyl, and methadone. This novel study provided a new strategy for the treatment of drug addiction [53]. Similarly, synthesized RNA oligonucleotides were transferred to MSCs in order to produce miR-143-rich exosomes, inhibiting the migratory potential of osteosarcoma cells [54]. Akt was transfected into umbilical cord-derived MSCs by using an adenovirus transfection system that improved cardiac function in animals treated with modified exosomes [55]. In addition, Pascucci reported that MSCs can acquire strong antitumor properties after incubation with paclitaxel (PTX), including the uptake of high drug doses followed by release into exosomes, inhibiting tumor growth activity. This method provides the possibility of using MSCs for the development of drugs with a higher cell-target specificity [56]. Sterzenbach reported the usage of the evolutionarily conserved late-domain (L-domain) pathway as a mechanism for loading exogenous proteins into exosomes [57]. They labeled an intracellular target protein with a WW tag, which was recognized by the L-domain motifs on Ndfip1, resulting in the loading of the target protein into exosomes.

For better tissue-targeting and an enhanced exosomal therapeutic effect, surface modification of exosomes was recently attempted by many groups using gene transfection techniques. The conventional method for surface protein loading was the expression of a genetic fusion between the targeted peptide and a protein that natively localized on the exosomal surface, such as Lamp2 [50]. Similarly, Ohno and colleagues engineered donor cells to express the transmembrane domain of platelet-derived growth factor receptor fused to the GE11 peptide, which efficiently delivered let-7a miRNA to epidermal growth factor receptor- (EGFR-) expressing breast cancer cells [58]. Furthermore, Tamura modified the exosomal surface by a simple mixing of original exosomes and cationized pullulan through an electrostatic interaction of both substances, thus targeting injured liver tissue and enhancing the therapeutic effects [59].

The fate of nucleic acid cargos in target cells remains controversial. For example, Kanada et al. suggests that exosomes cannot deliver functional nucleic acids to target cells by detecting differential fates of transfection-loaded biomolecules (plasmid DNA (pDNA), mRNA, and siRNA) delivered to target cells [60].

### 4.3. MSCs as an Ideal Source of Exosomes for Drug Delivery

Despite the fact that the properties of natural MSC-derived exosomes are disputed and distinctive of different origins, the use of MSC-derived exosomes has been confirmed for the cell type-specific targeting of drug delivery as a better alternative because of several features. First, exosomes do not elicit acute immune rejection, and there is no risk for tumor formation [61]. Second, MSCs are efficient mass producers of exosomes, which can be manufactured large scale in culture [62], providing support for individualized therapy. Third, the safety of exosomes has been confirmed in vivo by different animal models [63, 64]. To achieve cell-specific targeted drug delivery, several studies have tested donor cells, loading methods, and therapeutic cargos of MSC-derived exosomes. Bone marrow stem cells are typically used as the donor cells, and miRNAs are typically used for therapeutic cargos (Table 2). A phase II-III study has also been processed by a group in Egypt, who hypothesized that intravenous infusion of cell-free umbilical cord blood-derived MSC microvesicles may reduce the inflammatory state, thus improving the *β*-cell mass and glycemic control in patients with type 1 diabetes (T1DM). However, these outcomes remain controversial, particularly in reference to dose responses. The data reported in several studies is highly dependent on the drug-loading of exosomes, not the quantity of exosomes.

## 5. Exosomes for Cell-Free Therapy

Currently, the use of exosomes as early diagnostic tools for various types of cancer is underway. In addition, the use of exosomes as diagnostics for prostate cancer is undergoing FDA-approved tests. While complexities surrounding the therapeutic potential of exosomes continue to unravel, several clinical trials (Table 3, data from http://clinicaltrials.gov) have been completed or are underway in order to evaluate this therapeutic potential. In these trials, largely modified exosomes were used rather than native exosomes.

## 6. Conclusion and Perspective

The therapeutic potential of exosomes presents exciting new avenues for intervention in many diseases. The ability instinct to transport genetic messages and to protect the cargos to natural preferential recipient cells has drawn a rapid rise in attention. Therefore, specialized journals and websites have been established to disseminate this continuously unraveling information. While various clinical trials are underway to evaluate the safety and effectiveness of exosomes as therapeutic targets, many issues still remain. Questions regarding how clinical-grade exosomes can be produced in quantity and how various loading and isolation strategies impact the potency of exosome-based drug delivery remain unanswered. Therefore, there is an urgent need to closely examine the following aspects of exosomes: (1) natural therapeutic potential; (2) biogenesis mechanism; and (3) circulation kinetics and biodistribution. There is still a long road ahead, from promising phenomenological observations to clinical applications.

## Figures and Tables

**Table 1:** Characterization of various extracellular vesicles.

	Exosomes	MVs	Apoptotic bodies
Size	30–100 nm	50–2000 nm	500–4000 nm
Surface markers (used most)	CD63	ARF6	TSP
	CD9	VCAMP3	C3b

**Table 2:** Studies focusing on MSC-derived exosomes, their loading methods, therapeutic cargos, and biological function.

Origin	Disease models	Biological function	Contributing factors	Artificial modification & loading methods	Articles
Mouse bone marrow-derived MSCs (BM-MSCs)	Breast cancer	Suppress angiogenesis and tumor progression	miR-16	/	Lee et al. [35]

Human adult liver stem cells (HLSCs)	Hepatoma	Transfer genetic information that interferes with the deregulated survival and proliferation	miR451, miR223, miR31, and so on	/	Fonsato et al. [33]

MSCs	Glioma	Reduce glioma xenograft growth in rat brain	miR-146b	Transfection and electroporation	Katakowski et al. [65]

Mouse BM-MSCs	Pancreatic adenocarcinoma	Inhibit in vitro tumor growth	Paclitaxel	Incubation	Pascucci et al. [56]

Human umbilical cord Wharton's jelly mesenchymal stem cells (hWJMSCs)	Bladder tumor	Downregulated phosphorylation of Akt protein kinase and upregulated cleaved caspase 3 during the process of antiproliferation and proapoptosis	Akt and caspase 3	/	Wu et al. [66]

MSCs	Liver injury	Induces an anti-inflammatory effect	Cationized pullulan	Incubation	Tamura et al. [59]

Mouse embryonic fibroblast (MEF) glioblastoma cells	/	Engineer exosomes containing Cre recombinase that have been used to identify functional delivery of exosomes across the blood-brain barrier to recipient neurons in the brain	Labeling of a target protein, Cre recombinase, with a WW tag	Transfection	Sterzenbach et al. [57]

BM-MSCs	Colonic carcinoma gastric carcinoma	Promote VEGF and CXCR4 expression in tumor cells through ERK1/2 and p38 MAPK pathways	/	/	Zhu et al. [67]

Mouse immature dendritic cells (imDCs)	Breast cancer	Target tumor therapy	Doxorubicin	Electroporation transfection	Tian et al. [50]

Human umbilical cord-derived MSCs (hucMSCs)	Acute myocardial infarction	Accelerate endothelium cell proliferation, migration, and vessel formation	Platelet-derived growth factor D (PDGF-D)	Transfection	Ma et al. [55]

BM-MSCs	Status epilepticus (SE)	Minimize the adverse effects of SE in the hippocampus and prevent SE-induced cognitive and memory impairments	/	/	Long et al. [68]

BM-MSCs	Ameliorate inflammation-inducedpreterm brain injury	Ameliorate inflammation-induced neuronal cellular degeneration, reduce microgliosis, and prevent reactive astrogliosis	/	/	Drommelschmidt et al. [69]

hucMSCs	PBMCs	Immunosuppressive function	/	/	Monguio-Tortajada et al. [70]

Cardiomyocyte progenitor cell (CMPC) MSCs	Human microvascular endothelial cells (HMECs) and human umbilical vein endothelial cells (HUVECs)	Proangiogenic effects	Extracellular matrix metalloproteinase inducer (EMMPRIN)	/	Vrijsen et al. [29]

MSCs	Breast cancer	Deliver antagomiRs to chemosensitize the BCCs and to prevent dormancy	Anti-miR-222/-223	Transfection	Bliss et al. [71]

BM-MSCs	PBMCs	Incorporated by monocytes, lowering the effect on lymphocyte populations	/	/	Di Trapani et al. [37]

BM-MSCs	Hepatocellular carcinoma, ovarian cancer, Kaposi's sarcoma	Induce in vitro cell cycle arrest and apoptosis or necrosis of different tumor cell lines and in vivo inhibit growth of established tumors	/	/	Bruno et al. [72]

BM-MSCs	Breast cancer	Induce dormant phenotypes through the suppression of a target gene, MARCKS, which encodes a protein that promotes cell cycling and motility	miR-23b	/	Ono et al. [34]

hucMSCs	Hepatitis C virus (HCV)	Suppress HCV RNA replication	let-7f, miR-145, miR-199a, and miR-221	/	Qian et al. [36]

BM-MSCs	Osteosarcoma	Suppress the migration of the 143B osteosarcoma cell line	miR-143	Transfection	Shimbo et al. [54]

**Table 3:** Clinical trials of exosome–based therapies.

Study title	Disease	Intervention	Phase	NCT
Effect of plasma-derived exosomes on intractable cutaneous wound healing: prospective trial	Ulcer	Autologous exosomes Rich plasma	Early phase 1	NCT02565264

Study investigating the ability of plant exosomes to deliver curcumin to normal and colon cancer tissue	Colon cancer	Curcumin delivery by exosomes	Phase 1	NCT01294072

Effect of microvesicles and exosomes therapy on *β*-cell mass in type I diabetes mellitus (T1DM)	Type I diabetes mellitus	Umbilical cord blood-derived MSC microvesicles	Phase 2 Phase 3	NCT02138331

Preliminary clinical trial investigating the ability of plant exosomes to abrogate oral mucositis induced by combined chemotherapy and radiation in head and neck cancer patients	Head and neck cancer Oral mucositis	Dietary Supplement: grape extract Drug: lortab, fentanyl patch, mouthwash	Phase 1	NCT01668849

Pilot study of metformin in head and neck cancer and its effect on proinflammatory cytokines and exosomes implicated in acute and chronic toxicity	Head and neck cancer	Radiation: external beam radiation therapy Drug: metformin hydrochloride Other: placebo	Early phase 1	NCT03109873

Phase II trial of a vaccination with tumor antigen-loaded dendritic cell-derived exosomes on patients with unresectable non-small-cell lung cancer responding to induction chemotherapy	Non-small-cell lung cancer	Tumor antigen-loaded dendritic cell-derived exosomes	Phase 2	NCT01159288

Clinical trial of tumor cell-derived microparticles packaging chemotherapeutic drugs to treat malignant pleural effusion	Malignant pleural effusion	Biological: tumor-derived microparticles Drug: cisplatin	Phase 2	NCT02657460
